# A common French-Italian laminopathy registry – update & future prospects

**DOI:** 10.1186/1750-1172-10-S2-O31

**Published:** 2015-11-11

**Authors:** Gisèle Bonne, Rabah Ben Yaou

**Affiliations:** 1 Sorbonne Universités, UPMC Univ Paris 06, INSERM UMRS974, CNRS FRE3617, Center for Research in Myology, Paris, France

## 

In front of the wide clinical and genetic heterogeneity of the laminopathies, the first task of the French Network on EDMD and other related nuclear envelope related diseases, has been to set up in 2000, a mutation database for *LMNA* and *EMD* mutations. We selected the Universal Mutation Database tool (UMD) developed by Christophe Beroud (http://www.umd.be,[1]) and set up the UMD-*LMNA* database that compiles genetic and associated main clinical features of both in-house identified cases, those submitted to us by other partners involved in *LMNA* gene analysis, and also all mutated subjects reported in the literature (http://www.umd.be/LMNA/). To date, the UMD-*LMNA* database comprises 510 different *LMNA* mutations identified in over 2700 individuals, out of which 60% presenting with a laminopathy of the striated muscles (cardiomyopathies+/-muscular dystrophies), 14% with a laminopathy affecting the adipose tissue (metabolic syndromes +/- partial lipodystrophies) and 5.2% with premature ageing syndrome.

For rare diseases, a major hurdle in clinical translation of basic science research results is the difficulty in identifying appropriate patient cohorts. Prospective data on patient clinical characteristics, specific biomarkers and outcome measures are also frequently unavailable. With the aim to obtain longitudinal clinical data on patients with laminopathy or emerinopathy (as well as asymptomatic carriers at the time of genetic diagnosis) and create corresponding “trial-readiness” patient registries, we set-up in 2013 a prospective patient registry, the OPALE registry (for “Observatoire des Patients Atteints de Laminopathie et Emerinopathie”). This registry will allow precise characterization of the disease natural history, identification of specific outcome measures and further evaluation of the prognostic value of various biomarkers. The overall goals of OPALE are *1)* a better follow-up and management of the patients and *2)* the identification of specific parameters to monitor treatments in the perspective of future clinical trials. OPALE has been initiated within 3 pilot centers (neuromuscular reference center at the Myology Institute, cardiology department of Cochin university hospital in Paris, neuropediatric department of Raymond Poincaré university hospital in Garches) and thanks to the strong links within the French Networks of “EDMD & other nuclear envelope related diseases”, OPALE is now progressively opened to other French reference centers, once the approvals of IRB/ethics committee and other regulatory authority have been obtained. To date, 78 patients have been included in this registry among 170 followed within the 3 pilot centers. Regular update of the collected data are planned. We plan to open the registry to international colleagues via our interaction with the Italian network for Laminopathies, with the TREAT-NMD networks as well as any other center interested in this initiative. We hope the OPALE registry will become rapidly an international interactive tool for the benefit of laminopathy and emerinopathy patients.

## References

[B1] Beroud C Hamroun D Collod-Beroud G Boileau C Soussi T Claustres M UMD (Universal Mutation Database): 2005 update Human mutation 2005 26 3 184 91 16086365 10.1002/humu.20210

